# Laryngeal schwannoma with extralaryngeal extension mimicking a thyroid tumour

**DOI:** 10.1259/bjrcr.20210089

**Published:** 2022-03-09

**Authors:** Ayako Mikoshi, Hiromi Edo, Tatsu Hase, Taishi Sakima, Kosuke Uno, Fumihisa Kumazawa, Kimiya Sato, Hiroshi Shinmoto

**Affiliations:** 1 Department of Radiology, National Defense Medical College, Tokorozawa, Japan; 2 Department of Otolaryngology, National Defense Medical College, Tokorozawa, Japan; 3 Department of Basic Pathology, National Defense Medical College, Tokorozawa, Japan

## Abstract

**Objective:**

A schwannoma is a common benign tumour that can arise anywhere in the body. When it occurs in an unusual location such as the larynx, its differentiation from other tumours can be challenging. Herein, we report a case of a laryngeal schwannoma with extralaryngeal extension that mimicked a thyroid tumour, focusing on its characteristic features on MRI.

**Methods:**

A 19-year-old male presented with a mass in the left side of the neck and hoarseness for 2 years. Endoscopy showed a submucosal mass in the laryngeal region. MRI found a well-defined solid mass in the thyroid gland, extending to the larynx through the lower edge of the thyroid cartilage. *T*_2_ weighted MRI showed slightly low signal intensity at the central part of the tumour and high signal intensity at the peripheral part of the tumour. Pre-operative imaging suggested that the tumour originated in the thyroid gland. Left thyroidectomy with tumour excision was performed; the tumour was diagnosed as a laryngeal schwannoma with extralaryngeal extension, compressing the thyroid gland. In retrospect, features such as the dumbbell-shape and known as ‘target sign’ on *T*_2_ weighted MRI were typical features of schwannoma. Additionally, the tumour’s extension pattern was similar to previous reports of laryngeal schwannomas with extralaryngeal extension.

**Conclusion:**

A large laryngeal schwannoma may extend outside the larynx with significant compression of the thyroid gland. Understanding the pattern of extension and familiarity with the features on MRI can improve the preoperative diagnosis accuracy.

## Background

Schwannomas are common benign tumours derived from Schwann cells in the peripheral nervous system.^1^ They can occur anywhere from head to toe; however, those in the larynx are rare, with an incidence of 0.1–1.5%.^1^ Due to the presence of symptoms such as hoarseness which facilitates early detection, laryngeal schwannomas are usually small. Those that grow in size and extend to the thyroid gland are extremely rare.^1,2^ Only three previous cases of laryngeal schwannomas with extralaryngeal extension have been reported^1,3,4^; therefore, little is known about their characteristics on MRI. We report the MRI findings of a laryngeal schwannoma with extralaryngeal extension, focusing on the pattern of extension and the MRI features in an effort to improve preoperative diagnostic accuracy.

## Case report

A 19-year-old male presented with a mass in the left side of the neck and hoarseness for 2 years. There was no family or other medical history relevant to the main complaint in this case. Laryngoscopy showed a submucosal mass in the laryngeal region. The tumour had compressed the left vocal cord.

**Figure 1.**
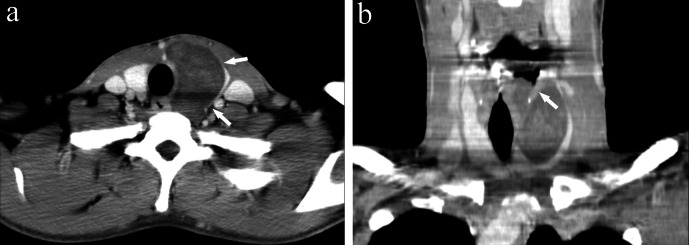
Contrast-enhanced (CT) images. (a) CT demonstrates a solid tumour showing a “beak sign” in the thyroid gland (arrows). (b) The tumour is present both in and around the larynx through the lower edge of the thyroid cartilage (arrow).

**Figure 2.**
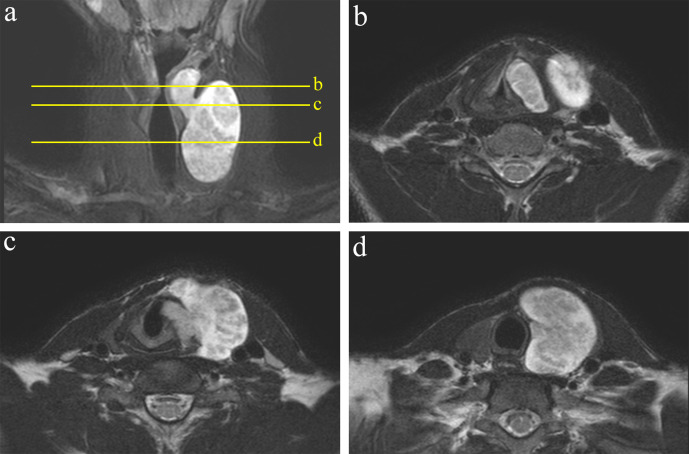
Magnetic resonance images. (a) Coronal fat-suppressed T2-weighted image shows a dumbbell-shaped tumour extending to the paralaryngeal region through the lower edge of the thyroid cartilage. The yellow lines indicate slice position for Figures b-d. (b–d) The tumour has high peripheral signal intensity and low central signal intensity on T2-weighted images.

**Figure 3.**
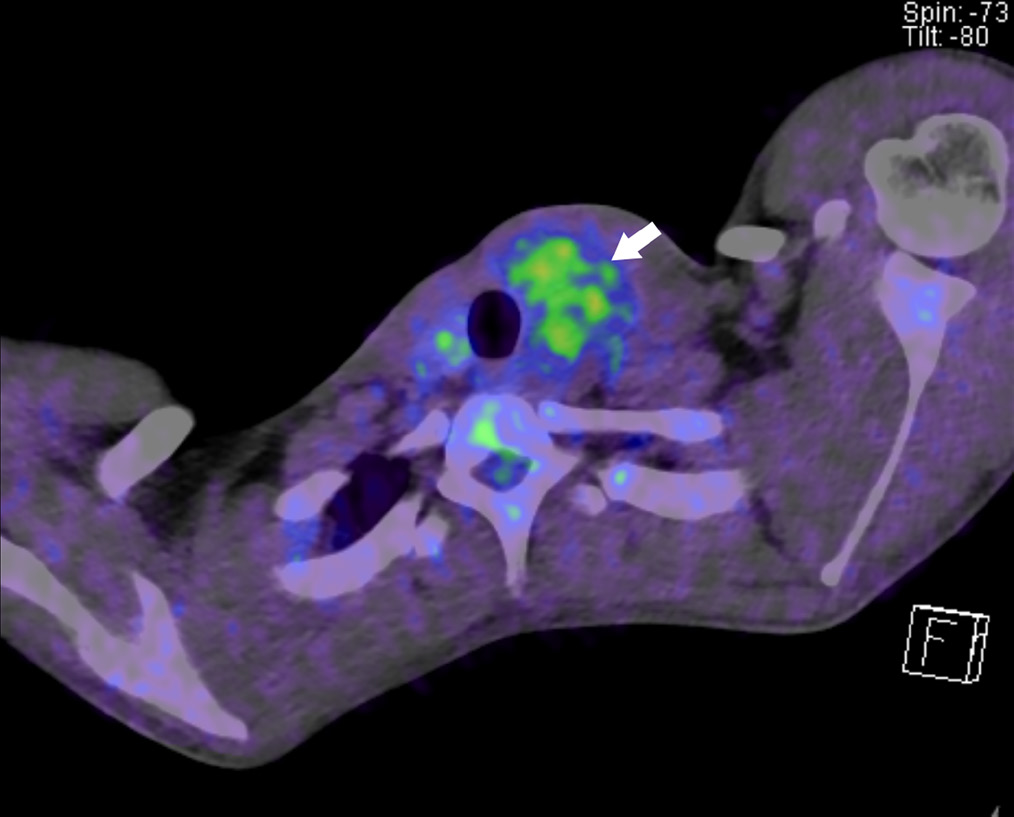
FDG-positron emission tomography. Mild to moderate FDG uptake (arrow) in the tumour is observed. The maximum standardized uptake value is 3.18. FDG, 18F-flurodeoxyglucose.

CT demonstrated a well-defined solid tumour 5.6 cm in size in the left lobe of the thyroid gland (Figure 1). The tumour extended to the larynx but did not invade the surrounding tissues, and there were no signs of cartilaginous destruction. Coronal fat-suppressed *T*_2_ weighted MRI (*T*_2_WI) showed a well-defined mass in the left lobe of the thyroid gland extending to the paralaryngeal region through the lower edge of the thyroid cartilage (Figure 2a). On *T*_2_WI, the tumour had a lesional pattern, with a central area of slightly low intensity surrounded by a hyperintense rim (Figure 2b-d). ^18^F-fludeoxyglucose (FDG)-positron emission tomography (PET) showed mild to moderate FDG uptake in the tumour (Figure 3). Pre-operative diagnosis proved to be difficult, as such, we considered that a non-specific mass had originated in the thyroid gland.

**Figure 4.**
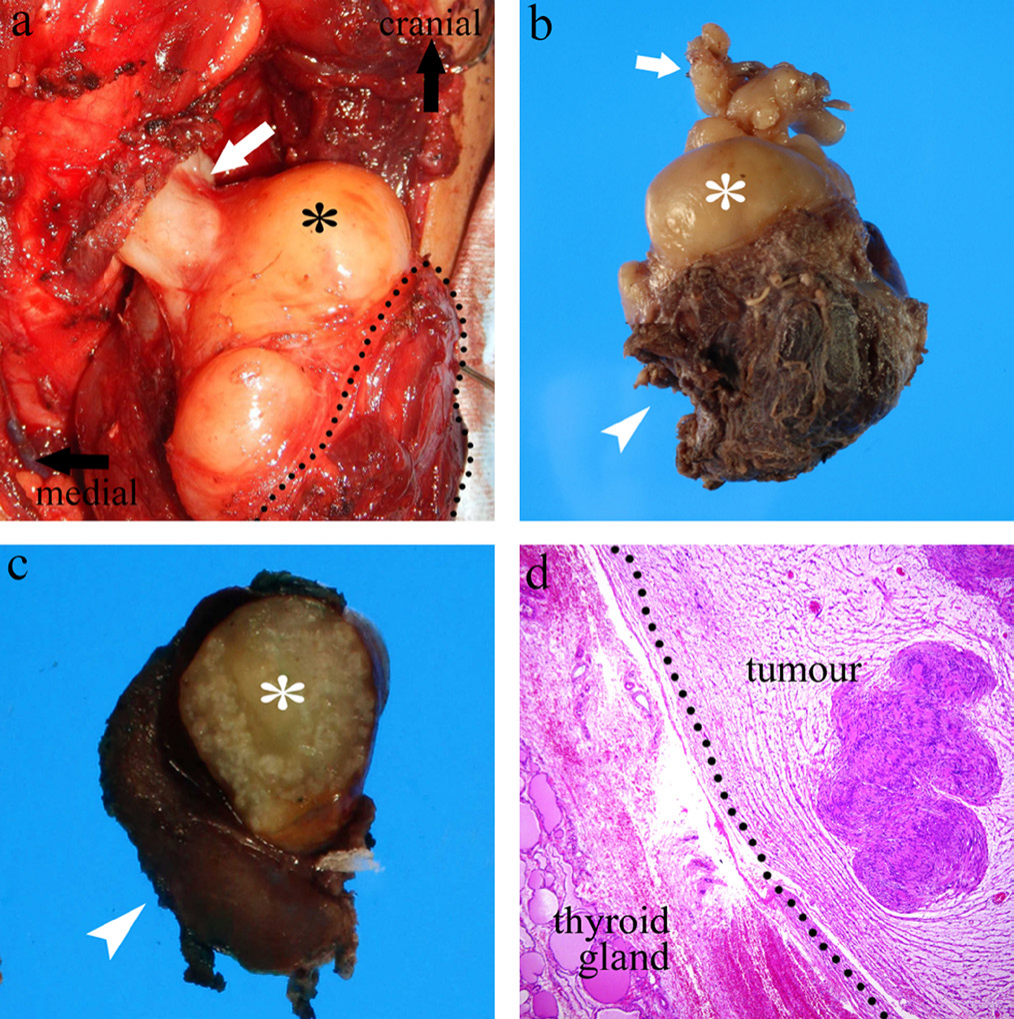
Intraoperative photograph and histopathological features of a resected specimen. (a) The tumour (asterisk) is partially attached to the thyroid gland (dotted line) and penetrates the larynx through the space behind the cricothyroid muscle (white arrow). (b) Gross image reveals a yellowish-white, solid, dumbbell-shaped tumour (asterisk) is partially attached to the thyroid gland (arrowhead). The white arrow indicates the part of the tumour that extends into the larynx. (c) Grossly, the tumour has a well-defined contour (asterisk) without invasion of the thyroid gland (arrowhead). (d) Haematoxylin and eosin staining (×40 magnification) shows two different growth patterns (Antoni A and Antoni B). There is no obvious invasion of the thyroid gland.

The tumour was pathologically diagnosed as a schwannoma by open biopsy, and tumour resection and left thyroidectomy were performed. Intraoperatively, the extension of the tumour through the space behind the cricothyroid muscle to the larynx and surrounding tissue was apparent (Figure 4a). Macroscopically, a yellow-white solid dumbbell-shaped tumour attached to the thyroid gland was observed (Figure 4b, c). Microscopically, the tumour had two different growth patterns (Antoni A and Antoni B) (Figure 4d). Immunohistochemically, tumour cells were positive for S-100 protein. The tumour strongly compressed the left lobe of the thyroid gland from the dorsal side, but there was no apparent invasion of the thyroid gland. At the 8 month post-operative follow-up, the hoarseness had lessened, vocal cord movement was good, and no recurrence was observed on MRI.

**Figure 5.**
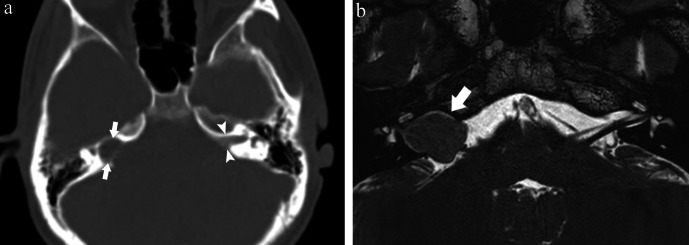
(a) Computed tomography (CT) component of a positron emission tomography (PET)-CT shows dilatation of the right internal auditory canal (white arrows) compared to the left internal auditory canal (white arrowheads). (b) Magnetic resonance cisternography reveals a vestibular schwannoma located between the internal auditory canal and the cerebellopontine angle (white arrow).

After the laryngeal schwannoma was identified, the CT portion of the PET-CT was reviewed again, finding dilatation of the right internal auditory canal (Figure 5a). A vestibular schwannoma was suspected, and a subsequent MRI was performed. MRI revealed a vestibular schwannoma located from the right internal auditory canal to the cerebellopontine angle (Figure 5b).

## Discussion

Laryngeal schwannomas can extend beyond the larynx as their size increases.^1^ In this case, since the main tumour component occupied the thyroid region, it was difficult to exclude invasion of the thyroid gland, and pre-operatively, we considered the mass originating in the thyroid gland. Retrospectively, a better understanding of the pattern of extension and imaging findings may have helped us to make a differential diagnosis of laryngeal schwannoma with extralaryngeal extension.

Laryngeal schwannomas are commonly derived from the internal branch of the superior laryngeal nerve.^1^ In previous reports,^1,2^ the mean size of laryngeal schwannomas was 2.5 cm. In our case it was 5.6 cm, suggesting that the tumour extended beyond the larynx. The pattern of tumour spreading in our case was the same as that in a previous report.^1^ Only 3 of the 74 laryngeal schwannomas reviewed by Tulli et al^5^ extended beyond the larynx; 2 of the 3 were neurofibromatosis Type 2 (NF2)^3,4^ and 1 was a schwannomatosis.^1^ In the present case, there was no family history suggestive of NF2 or schwannomatosis, but additional MRI showed a vestibular schwannoma located from the internal auditory canal to the cerebellopontine angle. Our case consisted of a pathologically confirmed non-dermal schwannoma and unilateral vestibular schwannoma, suggested schwannomatosis as the clinical diagnosis.^6^ All cases of laryngeal schwannoma with extrapharyngeal extension, including the present case, were associated with NF2 or schwannomatosis.

Target sign is a well-known characteristic finding of schwannoma on MRI; the central area of schwannoma showing a low signal intensity on *T*_2_WI corresponds to Antoni A, and the marginal area showing a high signal intensity on *T*_2_WI corresponds to Antoni B.^5^ Based on the MRI findings, the target sign on *T*_2_WI was also observed in our case. Compared with CT, MRI can provide a more detailed depiction of internal features, such as the cellularity of the tumour.^5^ The heterogeneous appearance of schwannomas may be attributed to degeneration and the plethora of cellular components such as cystic and xanthomatous lesions.^7^ Small schwannomas tend to have homogenous contrast enhancement after gadolinium administration, whereas larger lesions have more heterogeneous enhancement.^5^ FDG uptake by schwannomas is variable, so it is difficult to differentiate between benign and malignant tumours.^8^ We emphasize the importance of ascertaining the presence of the tumour extension via imaging. The laryngeal tumour in our case had the typical features of a schwannoma despite its atypical location.

Schwannomas are known to have a dumbbell-shaped morphology, as found in spinal schwannomas and vestibular schwannomas.^8–10^ These dumbbell-shaped schwannomas are characterized by schwannomas arising in a narrow bony foramen and extending outside to form a larger mass than the site of origin. In this case, although the schwannoma did not originate from a narrow bony foramen, it did originate from a narrow space in the larynx and had a dumbbell-shaped morphology.

When a contiguous tumour with no destructive changes in the surrounding area is found both inside and outside the larynx and its characteristics resemble those of schwannomas found elsewhere, the differential diagnosis should include laryngeal schwannoma. Knowing that laryngeal schwannomas can extend from a loose laryngeal structure to the extralaryngeal region is essential. Keeping these features in mind will facilitate accurate preoperative imaging-based diagnosis of laryngeal schwannomas with extralaryngeal extension.

## Conclusion

A large laryngeal schwannoma may extend to the extralarynx with significant compression of the thyroid gland and may be misdiagnosed as a thyroid tumour. Understanding its pattern of extension and familiarity with its characteristics on MRI will improve pre-operative diagnostic accuracy.

## Learning points

- A laryngeal schwannoma may extend outside the larynx with significant compression of the thyroid gland without destructive bone changes.
- When *T*_2_ weighted MRI shows a characteristic 'target sign', schwannoma should be considered a differential diagnosis even if the mass is in an uncommon location, such as the larynx.
- All cases of laryngeal schwannoma with extralaryngeal extension reported so far have been NF2 or schwannomatosis.
- If NF2 or schwannomatosis is suspected, a previous PET-CT, if available, will provide an opportunity to detect other schwannomas by confirming enlargement of the internal auditory canal or intervertebral foramen with the CT component.
